# Endurance Training Counteracts the High-Fat Diet-Induced Profiling Changes of ω-3 Polyunsaturated Fatty Acids in Skeletal Muscle of Middle-Aged Rats

**DOI:** 10.3389/fphys.2019.00971

**Published:** 2019-07-30

**Authors:** Ting Li, Ding-Guo Ruan, Zhen-Mao Lin, Tai-Yang Liu, Kou Wang, Xiao-Yang Xu, Rui Duan

**Affiliations:** Laboratory of Exercise Biochemistry and Nutrition, School of Sports Science, South China Normal University, Guangzhou, China

**Keywords:** endurance training, high-fat diet, ω-3 polyunsaturated fatty acid, skeletal muscle, lipidomic profile

## Abstract

**Purpose:**

To investigate the effects of endurance training on the content of ω-3 polyunsaturated fatty acids (PUFAs) and their distribution among lipid classes in skeletal muscle in middle aged, high-fat diet fed rats.

**Method:**

Thirty 10-month old male Sprague Dawley (SD) rats were assigned to four groups. Two groups of rats remained sedentary and were fed chow diet (C group), or high-fat diet (H group), respectively. The other two groups of rats were subjected to endurance training while maintaining their chow diet (EC group), or high-fat diet (EH group). After 16 weeks endurance training and/or diet intervention, the content of ω-3 PUFAs and ω-3 PUFA-containing lipids in rat soleus muscle were analyzed by lipidomics.

**Results:**

Rats fed a high-fat diet exhibited decreased overall amount of ω-3 PUFAs in soleus muscle, while endurance training preserved the total amount of ω-3 PUFAs. Both the endurance training and high-fat diet alone changed the profiles of ω-3 PUFAs in different lipid classes. Specifically, the amount of triacylglycerol (TG), lysophosphatidylcholine (LPC), phosphatidylcholine (PC), and phosphatidylglycerol (PG) containing ω-3 PUFAs in soleus muscle was increased by endurance training, but the amount of lysophosphatidylenthanol (LPE), lysophosphatidylinositol (LPI), lysophosphatidylserine (LPS), cardiolipin (CL), phosphatidic acid (PA), and phosphatidylinositol (PI) was decreased. The high-fat diet induced a decrease of ω-3 PUFAs in TG, LPE, LPS, CL, platelet activating factor (PAF), PC, phosphatidylethanolamine (PE), and phosphatidylserine (PS), and an increase in LPC, LPI, PA, and PG. In addition, the effects of the endurance training on ω-3 PUFAs in skeletal muscle was also evident in high-fat diets fed rats, which counteracts the profiling changes caused by high-fat diet feeding.

**Conclusion:**

The beneficial effects of endurance training on skeletal muscle may be achieved to some extent through recovering the content of ω-3 PUFAs that has been decreased by high-fat diet feeding.

## Introduction

The detected members of lipid family in the organisms have exceed 180,000 different species (Dehairs et al., 2015). The functions of lipids include signaling, storing energy, and acting as components of bio-membranes, or precursors of steroid hormones. Lipids also participate in the process of cell apoptosis, signal transduction, infection and immune response. Strong evidences show that the metabolism of certain lipids is closely associated with insulin resistance (IR), diabetes, cancer, Alzheimer’s and cardiovascular disease (Hulver and Lynis, 2004; Jones et al., 2010; Han et al., 2011; Nomura and Cravatt, 2013; Kitessa and Abeywardena, 2016; Harari et al., 2017; Waldeyer et al., 2017). However, lipids containing ω-3 polyunsaturated fatty acids (PUFAs) are widely accepted to be beneficial for health (Harris, 1997; Mori et al., 2000; Shearer et al., 2012; Miller et al., 2014; Calder, 2015; Levental et al., 2017), which improve insulin sensitivity and decrease the fat deposition of skeletal muscle (Smith et al., 2010; Shearer et al., 2012; Sadeghi et al., 2016). ω-3 PUFAs are a family fatty acids identified by the first double bond positioned at the third carbon atom with respect to the methyl end, mainly including α-linolenic (ALA, 18:3ω-3), eicosapentaenoic acid (EPA, 20:5ω-3), docosapentaenoic acid (DPA, 22:5ω-3), and docosahexaenoic acid (DHA, 22:6ω-3). ω-3 PUFAs usually incorporate into GPs (ω-3 PUFA-containing GPs) in the bio-membrane and thus improves the fluidity of the membrane and the function of inserting proteins, such as insulin receptors. ω-3 PUFAs also directly activates G protein coupled receptors 120 (GPR120), which in turn mediates anti-inflammatory effects on macrophages and the insulin sensitizing effects on adipocytes (Oh et al., 2010).

Excessive fat intake induces ectopic lipid accumulation in skeletal muscle, and may consequently result in low insulin sensitivity, eventually IR and related metabolic syndromes (Li et al., 2018). Exercise is usually considered an effective way to decrease the ectopic lipid and increase insulin sensitivity (Erik and Mark, 2013; Marinho et al., 2019; Pattankuhar et al., 2019). However, endurance trained athletes with elevated level of triacylglycerol (TG) exhibited a higher insulin sensitivity compared to the obese patients with IR (Goodpaster et al., 2001). To explain this so-called “athletes’ paradox,” a subsequent study had been conducted and showed that levels of diacylglycerol (DG) and free ceramides, instead of TG, are more likely associated to insulin sensitivity (Amati et al., 2011). Later, researchers found that not all higher DG and ceramide levels result in IR, indicating that fatty acid composition of the accumulated lipids (lipid species) may be an important factor for high-fat diet-induced low insulin sensitivity and subsequent metabolic syndromes (Kitessa and Abeywardena, 2016). On the other hand, interests in the effects of exercise on the fatty acid composition of the lipid in tissues are increasing. Individual fatty acids play roles in tissue metabolism and signal transduction, such as the fatty acid composition of GPs (particularly ω-3 PUFAs) and insulin signaling (Borkman et al., 1993; Juárez-López et al., 2013). Despite of these studies, how endurance training or high-fat diet feeding affects the overall profiles of ω-3 PUFAs in skeletal muscle, and more specifically, the interactive effects of high-fat diet feeding and endurance training on ω-3 PUFAs remain undetermined. In this report we extend these studies to investigate the individual and interactive effects of endurance training and high-fat diet on the contents and distribution of ω-3 PUFAs in rats skeletal muscle, which may provide novel insights into the mechanism of enhanced insulin sensitivity with endurance training.

## Materials and Methods

### Animals

All animal experiments were conducted in accordance with the national guidelines of laboratory animal care and approved by the Ethics Committee of South China Normal University (SCNU). Ten-week-old male Sprague Dawley (SD) rats, purchased from Guangdong Medical Laboratory Animal Center, were housed in the animal lab of SCNU at 24 ± 1°C, 45–55% humidity and 12/12 light circle with free access to chow diet and water till they were 10-month old. The rats were assigned to sedentary group and exercise group according to the performance of 1-week treadmill exercise. And then, the two groups of rats were randomly divided into chow diet group and high-fat diet group, respectively. Eventually, the rats were assigned to four groups, including control group (C), high-fat diet group (H), endurance training with chow group (EC), and endurance training with high-fat diet group (EH). Each group includes 6–8 rats, and were kept 2 in one cage. During the entire experiment, the food consumption was recorded every day and the body weights were measured every week.

### Diets

The rats were fed for 16 weeks with the chow or high-fat diet after they are proper grouped. The chow diet was purchased from Guangdong Medical Laboratory Animal Center, and met the national standard for rat feed (3850 kcal/kg, 10% calorie from fat). And the high-fat diet was lab formulated (5% sucrose, 15% lard, 15% yolk powder, 0.5% sodium cholate, 1% cholesterol, and 63.5% chow diet) to provide 55% of total energy (5310 kcal/kg) from fat, and custom-made by Guangdong Medical Laboratory Animal Center. The fatty acid composition of the two diets was listed in Table 1.

**TABLE 1:** The fatty acid composition of the chow diet and high-fat diet.

	**Chow (%)**	**HFD (%)**		**Chow (%)**	**HFD (%)**
FA 11:0	3.13	6.18	FA 18:2	24.40	9.81
FA 12:0	0.73	1.45	FA 18:3	0.88	0.37
FA 16:0	16.53	14.34	FA 20:4	15.46	12.68
FA 17:0	0.17	2.45	FA 20:5	3.10	1.65
FA 18:0	2.94	20.74	FA 22:5	1.63	1.38
FA 19:0	0.01	1.03	FA 22:6	17.62	15.24
FA 18:1	10.20	10.54	OTHER	3.20	2.16

### Training Protocol

Exercise training was conducted using a treadmill designed for lab rats. Prior to endurance training protocol, the rats were habitual to treadmill running for 2 weeks till the running speed reached 18 m/min and the training time reached 1 h. Thereafter, the training groups was subjected to endurance training on a treadmill for 1 h at 18 m/min, 6 days per week, for 16 weeks. The sedentary groups were placed in resting treadmill at the same time as training groups. The rats were sacrificed around 36 h after the last training session.

### Insulin-Tolerance Test (ITT)

After 12 h fasting, the fasting blood glucose was measured using a glucometer (Accu-Chek, Roche, Switzerland). And then the rats were given one injection with a single 0.5 U/kgBW dose of insulin. The blood glucose was measured again at 30, 60, 90, and 120 min after injection, and the data were recorded and analyzed later for the insulin sensitivity.

### Lipid Analysis

#### Nomenclature

The lipids are classified, named, and the abbreviations are used according to LIPID MAPS^1^ : FA, fatty acyls; GL, glycerolipids; GP, glycerophospholipids; MG, monoadylglycerols; DG, diacylglycerol, TG, triacylglycerol; NEFA, non-esterified fatty acids; CL, cardiolipin; PA, phosphatidic acids; PAF, platelet activating factor; PC, phosphatidylcholine; PE, phosphatidylethanolamine; PG, phosphatidylglycerol; PI, phosphatidylinositol; PS, phosphatidylserine; and their respective lyso-species (LPA, lyso-PA; LPC, lyso-PC; LPE, lyso-PE; LPG, lyso-PG; LPI, lyso-PI; and LPS, lyso-PS). Lipid species were annotated according to their molecule composition as follows: lipid class (carbon atoms in the fatty acid: double bonds in the fatty acid), for example, PE(22:6_22:6). The letters “e” and “p” following numbers of double bond refer to ether and plasmalogen, respectively.

#### Lipid Extraction From Tissue Samples

All animals were sacrificed and the soleus on both side of each rat was obtained and frozen in liquid nitrogen, and the samples were stored at −80°C. Exactly weighed 50 mg of right soleus was treated with MTBE/CH_3_OH following the modified Folch method for lipid extraction (Matyash et al., 2008; Byeon et al., 2011). Briefly, the samples were mixed with 600 μL MTBE, 300 μL CH_3_OH and 300 μL H_2_O, followed by ultrasonic extraction for 30 min, and then centrifugation at 1000 × *g* for 15 min. The upper organic layer was taken into a new tube and blow-dried by Nitrogen. The final dried powder was re-dissolved into 400 μL CH_3_OH/isopropanol (1:1), centrifuged and the supernatant was obtained for the LC/MS analysis.

#### LC/MS Data Acquisition and Processing

Samples were analyzed by Thermo UltiMate 3000 LC, Q Exactive platform (Thermo Scientific) for lipidomics analysis in both positive and negative ion modes with a resolution of 70,000 at m/z = 250–1500 for MS and 17,500 for dd-MS^2^ experiments in a single acquisition. Calibration was performed by analyzing the pooled quality control (QC) samples, which was a mixture of an equal aliquot of all the samples in the experiment, at regular intervals throughout the run. Internal standards were not included in this untargeted approach. The overlay of the total ion chromatograms of the QC samples depicted excellent retention time reproducibility. And then, the individual samples were analyzed. MS data was used to monitor MG, DG, TG ions as ammonium and proton adducts; PAF, PC, and LPC as acetate adducts; and CL, FA, LPE, LPI, LPS, PA, PE, PG, PI, and PS as deprotonated anions. Data obtained from LC/MS were analyzed with Lipid Search (Thermo Scientific) software. Data post-processing and normalization were performed using Excel 2011 for mac. Lipids that contained ω-3 PUFAs were defined as those in which 22:6 or 20:5 fatty acids were detected. These are not the only possible ω-3 PUFAs in mammalian cells, but by far the most abundant. Furthermore, they are the only ones that could be definitively identified as ω-3 PUFAs using our lipidomic setup. Thus, the measurements of ω-3-containing lipids in this study may be slightly underestimated.

### Statistical Analysis

Principal component analysis (PCA) and partial least squares discrimination analysis (PLS-DA) were performed for group clustering and detection of the lipid outliers. Data analysis and Graphics are completed using R-project (Revolution Analytics, United States). The variable importance for the projection (VIP) statistics of PLS-DA were used for selecting significant variables responsible for group separation. Variables were selected as candidates when their VIP values were larger than 1.0 and the *p*-values were smaller than 0.05 by two-tailed *t*-test. Fold change (FC) comparison, expressed as | Log_2_(FC)| > 1.5, were also conducted to further confirm the outliers. Two-way analysis of variance (ANOVA; SPSS for mac) was used to test the effects of endurance training and/or high-fat diet, where all four groups were included for analysis at once (C, H, EC, EH); *post hoc* analysis were performed where there is an interaction effect between endurance training and high-fat diet by Bonferroni adjusted test. Body weights and food consumption were analyzed by two-way repeated measure ANOVA, and *post hoc* analysis were performed by Bonferroni adjusted test. All data are presented as means ± standard deviation. Statistical significance was set at *p* < 0.05.

## Results

A total of 1679 lipids were identified and quantified by LC/MS in positive mode and 679 lipids in negative mode, including FA, GL, GP, sphingolipids, sterol lipids, and prenol lipids. The detected lipids containing ω-3 PUFAs were listed in Supplementary Table S1.

### Weight Gain, Food Consumption, and Insulin Sensitivity of the Rats During the Experiment

**FIGURE 1:**
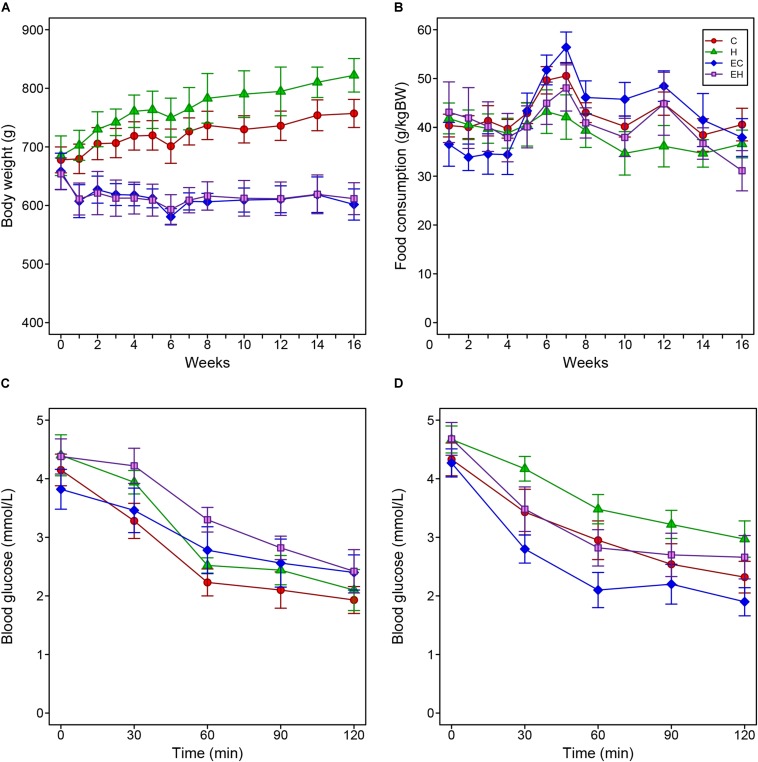
Changes of body weight **(A)** and food consumption **(B)** of the rats during the whole period of the experiment, and the results of injected insulin tolerance tests pre- **(C)** and post-intervention **(D)**.

Figures 1A,B show body-weight changes and food consumption among all experimental groups. Two-way repeated measure ANOVA analysis showed the body weights of C and H group significantly increased during the entire experiment, while both EC and EH groups kept their the body weight steady (Figure 1A). In Figure 1B, there were no significant differences of food intake between the four groups. However, considering the high-fat diet contains more calories than the chow diet (5310 kcal/kg vs. 3850 kcal/kg), the rats in H and EH groups actually took in more calories than the rats fed chow diet, which may be the reason that high-fat diet fed rats gained more weight. Additionally, in our experimental settings, the high-fat diet did not cause IR despite an decreased insulin sensitivity, endurance training significantly enhanced insulin sensitivity in both EC and EH group (Figures 1C,D).

### Multivariate Statistical Analysis of the Lipidomic Data

**FIGURE 2:**
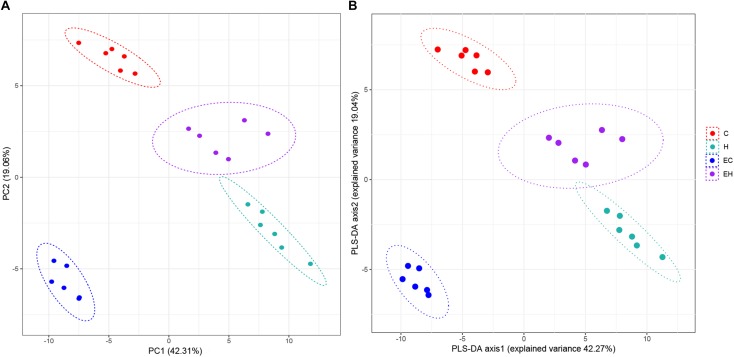
Plot of PCA **(A)** and PLS-DA **(B)** scores.

Unsupervised PCA and supervised PLS-DA (Figure 2) were performed with data obtained by LC/MS. VIP values, *t*-test *p*-values and FCs of the lipids were listed in the Supplementary Table S2, and the heatmap of top 25% lipids species were showed in Supplementary Figure S1. As shown in Figure 2, both of the results of PCA and PLS-DA showed clearly groups of the rats, which was consistent with their experimental groups.

### Effects of Endurance Training and/or High-Fat Diet on the Total Content of ω-3 PUFAs and ω-3 PUFA-Containing Lipid Categories

**FIGURE 3:**
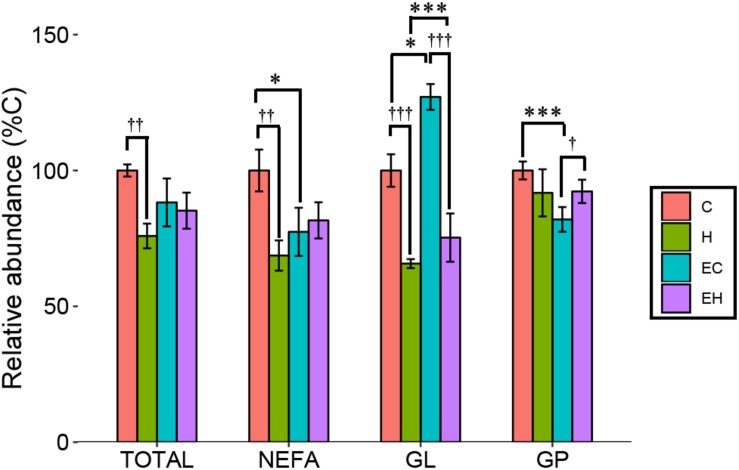
Total content of ω-3 PUFAs and ω-3 PUFA-containing lipid categories affected by endurance training and/or high-fat diet: ^*^p < 0.05 for endurance training effect; ^∗∗∗^*p* < 0.001 for endurance training effect; †*p* < 0.05 for high-fat diet effect; ††*p* < 0.01 for high-fat diet effect; †††*p* < 0.001 for high-fat diet effect; lines with sticks represent an interaction effect. Data are means ± SD.

In the present study, ω-3 PUFAs mainly exist as NEFAs, or components of GLs and GPs. Figure 3 shows changes of total ω-3 PUFAs and ω-3 PUFA-containing lipid categories (NEFA, GL, and GP) in the experimental groups. Two-way ANOVA analysis showed that there were interaction effects between the endurance training and high-fat diet feeding on total ω-3 PUFAs (*p* = 0.018) and ω-3 PUFA-containing lipids (*p* = 0.006 for NEFA; *p* = 0.002 for GL; *p* = 0.003 for GP). *Post hoc* analysis showed a significant decrease on the total content of ω-3 PUFAs in skeletal muscle by high-fat diet feeding (H vs. C, *p* = 0.001) while there is no significant effect of endurance training on total ω-3 PUFAs amount (E vs. C; H vs. EH). Further analysis in lipid categories showed that the content of ω-3 PUFA-NEFAs (EC vs. C; *p* = 0.011) and ω-3 PUFA-GPs (EC vs. C; *p* < 0.001) were decreased by endurance training, but no significant effects were observed in the high-fat diet fed rats (H vs. EH). Furthermore, a great increase by endurance training in ω-3 PUFA-GLs was shown in both chow diet (EC vs. C, *p* < 0.001) and high-fat diet fed rats (EH vs. H, *p* = 0.011). Thus, endurance training affected the distribution rather than the amount of ω-3 PUFAs in skeletal muscle. For the effects of high-fat diet alone, significant decrease of the ω-3 PUFAs in NEFAs (*p* = 0.001) and GLs (*p* < 0.001) was observed, but there is no statistically significant decrease in GPs (H vs. C).

### Effects of Endurance Training and/or High-Fat Diet on the Content of Non-esterified ω-3 PUFA

**FIGURE 4:**
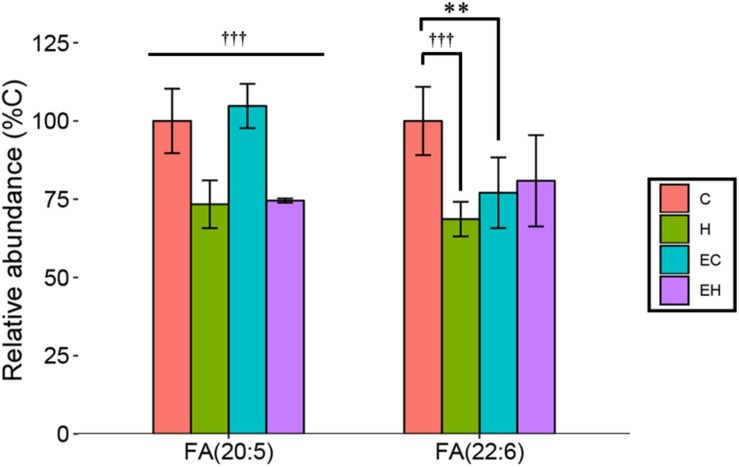
Changes of relative abundance of EPA and DHA in the skeletal muscle with endurance training and/or high-fat diet: ^∗∗^*p* < 0.01 for exercise effect; †††*p* < 0.001 for diet effect; straight lines represent the main effect of endurance training or high-fat diet; lines with sticks represent an interaction effect exists between endurance training and high-fat diet. Data are means ± SD.

In this study, we considered EPA [FA(20:5)] and DHA [FA(22:6)] the representatives of ω-3 PUFA as described in the section “LC/MS Data Acquisition and Processing.” They are mainly derived from the hydrolysis of TG and other lipids, reflecting the metabolism of the lipids. The results showed that the amount of EPA in skeletal muscle was significantly decreased by high-fat diet feeding (*p* < 0.001), but no significant effect of endurance training had been observed (Figure 4). Surprisingly, both endurance training (EC vs. C, *p* = 0.002) and high-fat diet feeding (H vs. C, *p* < 0.001) led to a great decrease in the relative amount of DHA in skeletal muscle. In addition, there was a trend that the content of DHA was increased by endurance training in high-fat diet fed rats (EH vs. H), indicating a possible synergistic effect of endurance training and high-fat diets fed. Eventually, the result suggested that the significant decrease of ω-3 PUFA-NEFAs in endurance training groups (Figure 3) was mainly attributed to the decrease of DHA.

### Effects of Endurance Training and/or High-Fat Diet on the Content of ω-3 PUFA-Containing Glycerolipid Classes and Species

**FIGURE 5:**
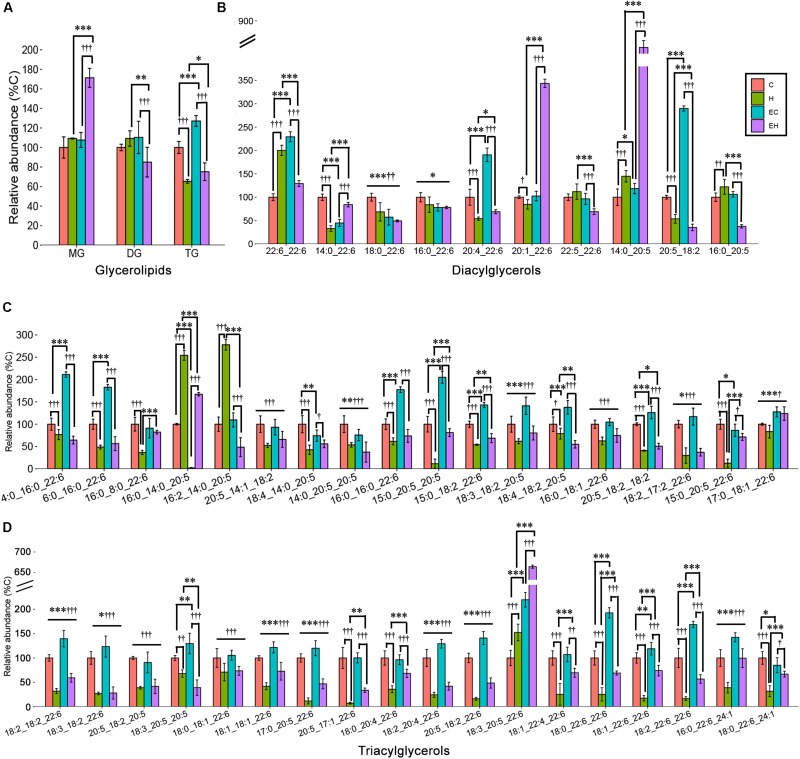
Effects of endurance training and/or high-fat diet on ω-3 PUFA-containing GL **(A)**, ω-3 PUFA-containing DG species **(B)** and ω-3 PUFA-containing TG species with statistical significance **(C,D)** in skeletal muscle: ^*^p < 0.05 for endurance training effect; ^∗∗^*p* < 0.01 for endurance training effect; ^∗∗∗^*p* < 0.001 for endurance training effect; †*p* < 0.05 for high-fat diet effect; ††*p* < 0.01 for high-fat diet effect; †††*p* < 0.001 for high-fat diet effect; straight lines represent the main effect of endurance training or high-fat diet; lines with sticks represent an interaction effect exists between endurance training and high-fat diet. Data are means ± SD.

Skeletal muscle is an organ that consumes energy to perform its essential function of locomotion. Given that TG is the most common energy source of skeletal muscle, DG and MG are the intermediates during hydrolysis of TG, we speculated that the endurance training would greatly affect the glycerolipid profiles. However, our analysis revealed that ω-3 PUFA-MGs and -DGs were not significantly affected by endurance training (EC vs. C) or high-fat diet feeding (H vs. C). Significant increase in -MGs (EH vs. H and EH vs. C, both *p* < 0.001) and decrease in -DGs (EH vs. H, *p* = 0.002; EH vs. EC, *p* < 0.001) were observed in EH group (Figure 5A). In addition, endurance training alone led to an increase in the content of ω-3 PUFA-TGs in the chow diet group (EC vs. C, *p* < 0.001). And, high-fat diet feeding alone induced a decrease in the relative abundance of ω-3 PUFA-TGs with or without endurance training (C vs. H, EC vs. EH, both *p* < 0.001, Figure 5A). For further species analysis, only DHA-MG of ω-3 PUFA-MGs was detected and analyzed in this study, we speculated that DHA was selectively preserved during the TG hydrolysis, especially in the EH group. Detailed analysis of DG species (Figure 5B and Supplementary Table S2) showed that the most affected DG species by endurance training were DG(20:5_18:2) in chow diet groups (EC vs. C) and DG(16:0_20:5) in high-fat diet groups (EH vs. H). DG(14:0_22:6) (H vs. C) and DG(20:5_18:2) (EH vs. EC) were significantly decreased by high-fat diet feeding. Endurance training and high-fat diet feeding synergistically increased DG(20:1_22:6) and DG(14:0_20:5) (EH vs. EC and EH vs. H, Figure 5B). Figures 5C,D shows the profiles of TG species which were significantly changed by the endurance training or high-fat diet feeding. Endurance training affected the content of most ω-3 PUFA-TG species (32 out of 36 species), although the FCs of 31 species were less than 1.5 (E vs. EC). Rats in H group exhibited a decreased content in most ω-3 PUFA-TGs compared with C group (33 out of 36 species). Interestingly, endurance training in EH group showed beneficial effects on some ω-3 PUFA-TGs when compared to H group.

### Effects of Endurance Training and/or High-Fat Diet on the Content of ω-3 PUFA Containing Glycerophospholipid Classes and Species

**FIGURE 6:**
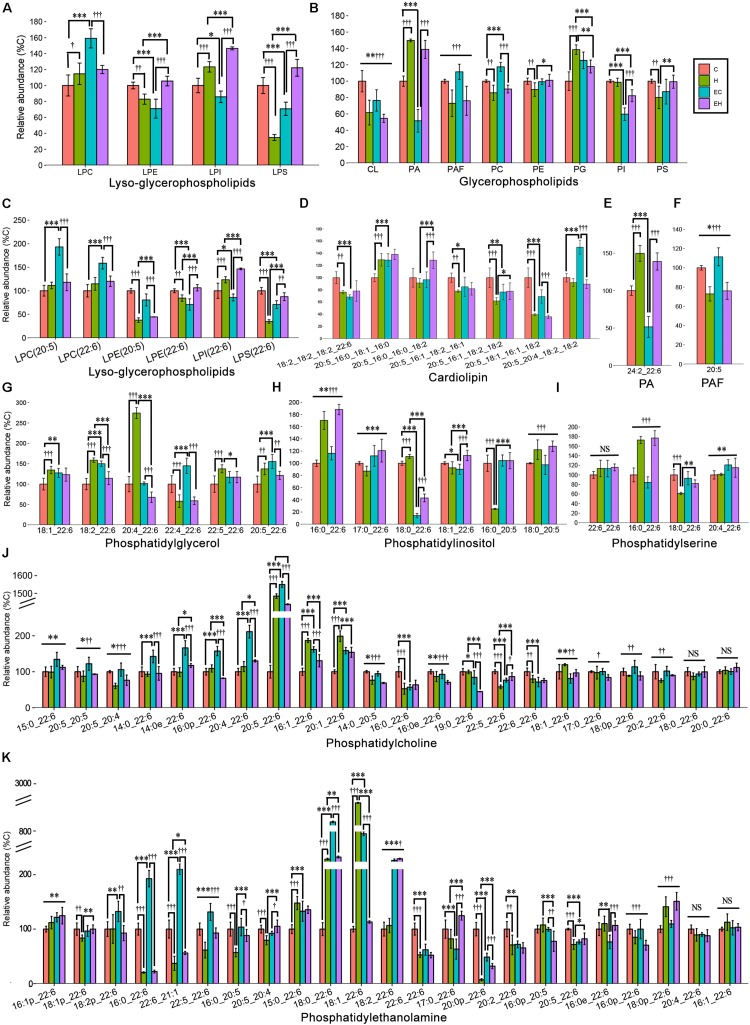
Lipidomic analysis of ω-3 PUFA-Glycerophospholipid classes **(A,B)** and the individual molecular species **(C–K)** in skeletal muscle: PA, phosphatidic acid; PAF, platelet-activating factor; ^*^p < 0.05 for endurance training effect; ^∗∗^*p* < 0.01 for endurance training effect; ^∗∗∗^*p* < 0.001 for endurance training effect; †*p* < 0.05 for high-fat diet effect; ††*p* < 0.01 for high-fat diet effect; †††*p* < 0.001 for high-fat diet effect; straight lines represent the main effect of endurance training or high-fat diet; lines with sticks represent an interaction effect exists between endurance training and high-fat diet. Data are means ± SD.

Figure 6 shows the different effects of endurance training and/or high-fat diet feeding on specific classes of GPs in the skeletal muscle (Figures 6A,B), including PA, PG, CL and PAF, and PC, PE, PI, PS and their lyso-species, and the species of each lipid class (Figures 6C–K). Generally speaking, endurance training alone showed effects with an increasing content of ω-3 PUFA-LPC (*p* < 0.001), -PC (*p* < 0.001), and -PG (*p* < 0.001), but a decreasing on the amount of ω-3 PUFA-LPE (*p* < 0.001), -LPI (*p* = 0.017), -LPS (*p* < 0.001), -CL (*p* = 0.003), -PA (*p* < 0.001) and -PI (*p* < 0.001). In addition, the ω-3 PUFA-GPs, including ω-3 PUFA-LPE (*p* = 0.001), -LPS (*p* < 0.001), -CL (*p* < 0.001), PAF (*p* < 0.001), PC (*p* = 0.001), PE (*p* = 0.009), and PS (*p* = 0.006) were decreased by high-fat diet feeding alone (Figures 6A,B). And others increased with high-fat diet feeding with an exception in ω-3 PUFA-PI. The detailed changes of GP species are also shown in Figures 6C–K. Among all the GP classes, PC and PE are the most abundant GPs in the cell membrane, and their fatty acid composition reflects the membrane biophysical properties. Endurance training resulted in an increase in ω-3 PUFA-PC level in chow diet fed rats and ω-3 PUFA-PE level in high-fat diet fed rats, while the high-fat diet feeding caused significant decreases both in ω-3 PUFA-PC and -PE (Figure 6B). The changes of their species seems to be inconsistent. Specifically, according to the calculated FC and *t*-test (Supplementary Table S2), PC (20:5_22:6), PE(18:0_22:6), PE(18:1_22:6), and PE(18:2_22:6) were significantly increased by endurance training alone (EC vs. C, Figures 6J,K), PC(20:5_22:6), PE(16:0_22:6), PE(18:0_22:6), PE(18:1_22:6) and PE(20:0p_22:6) were greatly affected by high-fat diet feeding alone (H vs. C, Figures 6J,K). In addition, when the two factor combined, endurance training greatly increased PE(18:2_22:6) and PE(20:0p_22:6), but decreased PE(18:1_22:6) (EH vs. H, Figure 6K), and high-fat diet feeding decreased PE(16:0_22:6), PE(18:0_22:6), PE(18:1_22:6) and PE(22:6_21:1) (EH vs. EC, Figure 6K).

## Discussion

The present study reported the detailed changes of ω-3 PUFAs and ω-3 PUFA-containing lipids with a 16-week endurance training and/or high-fat diet feeding in skeletal muscle of middle-aged rats. High-fat diet feeding induces a decrease in the total content of ω-3 PUFAs and most ω-3 PUFA-containing lipids, possibly due to low amount of PUFAs in the lard-based high-fat diet (Table 1), as the lipid composition of skeletal muscle is highly correlated with dietary fatty acids (Sato et al., 2012; Duan et al., 2014; Soni et al., 2016). Previous studies reported that endurance training increased the amount of ω-3 PUFAs in skeletal muscle (Andersson et al., 2000; Helge and Dela, 2003), which is inconsistent with our current study. The disagreements may be attributed to different muscle types or the exercise protocols. Some authors believed endurance training was mainly dependent on oxidative myofibers, so it should have a profound influence on ω-3 PUFAs in oxidative muscle (Andersson et al., 2000). However, other authors argued that endurance training affects glycolytic myofibers more because the oxidative myofibers already enriched with ω-3 PUFAs (Mitchell et al., 2004; Senoo et al., 2015). Consistent with the later concept, the present study showed that endurance training did not change the total content of ω-3 PUFAs in soleus which typically contains oxidative fibers. However, the endurance training affected the distribution of ω-3 PUFAs (ω-3 PUFA-containing lipids), which may be more important for their functions, such as the effects on insulin action. Secondly, Marina et al. (2011) investigated the effects of aerobic training in different intensities on the fatty acid composition of erythrocyte membranes, and reported that the EPA and DHA were decreased after low intensity training, while high intensity training resulted in a higher EPA and DHA level, and the EPA and DHA contents were still higher than low intensity group after 4 weeks of detraining. The reason may be that low intensity training leads to the transfer of ω-3 PUFAs out of the membrane and peroxidation of lipids, while high intensity of exercise may activate heat shock protein 70i (HSP70i) protein and enhance superoxide dismutase (SOD) activity.

In addition, our results show that endurance training leads to a significant increase in ω-3 PUFA-TGs, but does not significantly impact the contents of ω-3 PUFA-DGs and -MGs (Figure 5A). The increased ω-3 PUFA-TG level suggests a selectively synthesis of specific TG species by endurance training (Hu et al., 2010; Tomčala et al., 2010). DG is considered the most bioactive lipid that interacts with several signal pathways. We found that endurance training or high-fat diet feeding significantly affects the specific species of ω-3 PUFA-DG (Figure 5B), which may be mediated by regulation of monoacylglycerol acyltransferase (MGAT) and stearoyl-CoA desaturase 1 (SCD1) expression (Kawanishi et al., 2018). Furthermore, the results of our study show that both the ω-3 PUFA-PC and -PE contents were decreased by high fat diet, while endurance training exhibits a more significant effect on ω-3 PUFA-PC than -PE content (Figure 4B). This is consistent with Goto-Inoue et al. (2013), the authors reported that the high-fat diet resulted in a decrease of PC(18:2_22:6) of the extensor digitorum longus in rats. However, previous studies on the effects of exercise on skeletal muscle ω-3 PUFA-PC and -PE contents do not agree with each other. Mitchell et al. (2010) reported a decreased PC(18:0_22:6) in skeletal muscle, while Goto-Inoue et al. (2013) speculated that exercise may lead to an increase in the same lipid. These disagreements may be attributed to the different exercise types and intensities (Marina et al., 2011). Also, lysophosphatidic acid acyltransferase-3 (LPAAT3) may play a role in the exercised induced selective biosynthesis of ω-3 PUFA-GP. The mechanisms may include exercise activated AMPK/PGC-1α and PPARδ pathways, LPAAT3 expression, and biosynthesis of ω-3 PUFA-PA, which is the precursor of other ω-3 PUFA-GPs (Valentine et al., 2018).

In summary, high-fat diet feeding decreased overall ω-3 PUFAs contents in rats soleus muscle. Endurance training preserved the contents of ω-3 PUFAs and altered its distribution in different lipids. Given the beneficial functions of ω-3 PUFAs in insulin action, we speculated that endurance training may offset the high-fat diet feeding induced low insulin sensitivity through its effects on ω-3 PUFAs. However, further studies are needed to valid this speculation.

## Data Availability

The datasets generated for this study are available on request to the corresponding author.

## Ethics Statement

All animal experiments were conducted in accordance with the national guidelines of laboratory animal care and approved by the Ethics Committee of South China Normal University (SCNU).

## Author Contributions

TL, D-GR, Z-ML, T-YL, and KW performed the experiments. TL analyzed the data, interpreted the results of the experiments, prepared the figures, and drafted the manuscript. TL, X-YX, and RD edited and revised the manuscript, and approved the final version of the manuscript. TL and X-YX conceptualized and designed the research.

## Conflict of Interest Statement

The authors declare that the research was conducted in the absence of any commercial or financial relationships that could be construed as a potential conflict of interest.

## Footnotes

1 http://www.lipidmaps.org/data/classification/LM_classification_exp.php
