# Supplementary material for: Endurance Training Counteracts the High-Fat Diet-Induced Profiling Changes of ω-3 Polyunsaturated Fatty Acids in Skeletal Muscle of Middle-Aged Rats
Source: Front Physiol. 2019 Jul 30;10:971. doi: 10.3389/fphys.2019.00971 (PMC6683664; doi:10.3389/fphys.2019.00971)
Supplement: Supplementary file 1 [file Table_1.pdf]

Table 1: All ω-3 PUFA-containing lipids detected in this study and their molecular ion, Z-average molecular weight (CalcMz), Ion formula and retention time (Rt)

LipidIon	CalcMz	IonFormula	Rt	LipidIon	CalcMz	IonFormula	Rt
FA(22:6)-H	327.233	O2 H31 C22	1.662	TG(16:0_14:0_20:5)+NH4	842.723215	C53 H96 O6 N1	10.890
FA(22:6)-H	327.233	O2 H31 C22	1.661	TG(16:2_14:0_20:5)+NH4	838.691915	C53 H92 O6 N1	10.145
FA(22:6)-H	327.233	O2 H31 C22	1.6685	TG(20:5_14:1_18:2)+NH4	864.707565	C55 H94 O6 N1	10.240
FA(22:6)-H	327.233	O2 H31 C22	2.2735	TG(18:4_14:0_20:5)+NH4	862.691915	C55 H92 O6 N1	10.024
FA(22:6)-H	327.233	O2 H31 C22	3.1635	TG(15:0_18:2_20:5)+NH4	880.738865	C56 H98 O6 N1	10.795
FA(22:6)-H	327.233	O2 H31 C22	3.3325	TG(18:4_16:2_20:5)+NH4	886.691915	C57 H92 O6 N1	9.818
FA(22:6)-H	327.233	O2 H31 C22	4.3085	TG(18:3_18:2_20:5)+NH4	916.738865	C59 H98 O6 N1	10.404
FA(22:6)-H	327.233	O2 H31 C22	4.354	TG(18:4_18:2_20:5)+NH4	914.723215	C59 H96 O6 N1	10.217
FA(22:6)-H	327.233	O2 H31 C22	4.3805	TG(15:2_20:5_21:6)+NH4	910.691915	C59 H92 O6 N1	9.659
FA(22:6)-H	327.233	O2 H31 C22	5.34	TG(17:4_19:5_20:5)+NH4	908.676265	C59 H90 O6 N1	9.406
FA(22:6)-H	327.233	O2 H31 C22	5.2375	TG(20:5_18:2_18:2)+NH4	918.754515	C59 H100 O6 N1	10.562
FA(22:6)-H	327.233	O2 H31 C22	6.2695	TG(20:5_22:6_22:6)+NH4	1014.75452	C67 H100 O6 N1	10.102
FA(22:6)-H	327.233	O2 H31 C22	6.35	TG(15:0_20:5_22:6)+NH4	928.738865	C60 H98 O6 N1	10.385
FA(22:6)-H	327.233	O2 H31 C22	7.2705	TG(17:0_20:5_22:6)+NH4	956.770165	C62 H102 O6 N1	10.647
FA(22:6)-H	327.233	O2 H31 C22	7.2935	TG(20:5_17:1_22:6)+NH4	954.754515	C62 H100 O6 N1	10.488
FA(22:6)-H	327.233	O2 H31 C22	7.303	TG(20:5_18:2_22:6)+NH4	966.754515	C63 H100 O6 N1	10.323
FA(22:6)-H	327.233	O2 H31 C22	7.8665	TG(18:3_20:5_22:6)+NH4	964.738865	C63 H98 O6 N1	10.105
FA(22:6)-H	327.233	O2 H31 C22	7.8745	LPC(20:5)+HCOO	586.315046	C29 H49 O9 N1 P1	1.458
FA(22:6)-H	327.233	O2 H31 C22	8.7755	LPC(22:6)+HCOO	612.330696	C31 H51 O9 N1 P1	1.636
FA(22:6)-H	327.233	O2 H31 C22	9.003	LPE(20:5)-H	498.262616	C25 H41 O7 N1 P1	1.465
FA(22:6)-H	327.233	O2 H31 C22	9.5135	LPE(22:6)-H	524.278266	C27 H43 O7 N1 P1	1.666
FA(22:6)-H	327.233	O2 H31 C22	9.3605	LPI(22:6)-H	643.288892	C31 H48 O12 N0 P1	1.264
FA(22:6)-H	327.233	O2 H31 C22	11.0665	LPS(22:6)-H	568.268096	C28 H43 O9 N1 P1	1.352
FA(22:6)-H	327.233	O2 H31 C22	11.1335	CL(18:2_18:2_18:2_22:6)-H	1495.96495	C85 H141 O17 P2	9.805
FA(22:6)-H	327.233	O2 H31 C22	11.3715	CL(20:5_16:0_18:1_16:0)-H	1423.96495	C79 H141 O17 P2	10.545

FA(22:6)-H	327.233	O2 H31 C22	12.6225	CL(20:5_16:0_16:0_18:2)-H	1421.9493	C79 H139 O17 P2	10.320
FA(22:6)-H	327.233	O2 H31 C22	12.8725	CL(20:5_16:1_18:2_16:1)-H	1417.918	C79 H135 O17 P2	9.872
FA(22:6)-H	327.233	O2 H31 C22	13.094	CL(20:5_16:1_18:2_18:2)-H	1443.93365	C81 H137 O17 P2	9.902
FA(22:6)-H	327.233	O2 H31 C22	13.3905	CL(20:5_18:1_16:1_18:2)-H	1445.9493	C81 H139 O17 P2	10.125
FA(22:6)-H	327.233	O2 H31 C22	13.61	CL(20:5_18:2_18:2_18:2)-H	1469.9493	C83 H139 O17 P2	9.950
FA(22:6)-H	327.233	O2 H31 C22	14.821	CL(20:5_20:4_18:2_18:2)-H	1493.9493	C85 H139 O17 P2	9.887
FA(22:6)-H	327.233	O2 H31 C22	14.98	PA(24:2_22:6)-H	827.559632	C49 H80 O8 N0 P1	7.401
FA(20:5)-H	301.217	O2 H29 C20	2.7845	PAF(20:5)+HCOO	628.325611	C31 H51 O10 N1 P1	1.077
OAHFA(22:6_18:2)-H	605.458	C40 H61 O4	6.985	PC(14:0_20:5)+HCOO	796.513411	C43 H75 O10 N1 P1	6.223
MG(22:6)+H	403.284	C25 H39 O4	2.7	PC(14:0_22:6)+HCOO	822.529061	C45 H77 O10 N1 P1	6.562
MG(20:5)+H	377.269	C23 H37 O4	2.3445	PC(14:0e_22:6)+HCOO	808.549796	C45 H79 O9 N1 P1	7.054
DG(22:6_22:6)+H	713.514	C47 H69 O5	7.674	PC(15:0_22:6)+HCOO	836.544711	C46 H79 O10 N1 P1	6.920
DG(22:6_22:6)+NH4	730.541	C47 H72 O5 N1	7.776	PC(16:0_22:6)+HCOO	850.560361	C47 H81 O10 N1 P1	6.996
DG(20:5_20:5)+H	661.483	C43 H65 O5	7.085	PC(16:0_22:6)+HCOO	850.560361	C47 H81 O10 N1 P1	7.255
DG(20:5_20:5)+NH4	678.509	C43 H68 O5 N1	7.0245	PC(16:0e_22:6)+HCOO	836.581096	C47 H83 O9 N1 P1	7.768
DG(14:0_22:6)+H	613.483	C39 H65 O5	7.784	PC(16:0p_22:6)+HCOO	834.565446	C47 H81 O9 N1 P1	7.552
DG(18:0_22:6)+H	669.545	C43 H73 O5	8.85	PC(16:1_22:6)+HCOO	848.544711	C47 H79 O10 N1 P1	6.510
DG(18:_22:6)+H	663.498	C43 H67 O5	7.617	PC(16:1_22:6)+HCOO	848.544711	C47 H79 O10 N1 P1	6.680
DG(14:0_22:6)+NH4	630.509	C39 H68 O5 N1	7.8005	PC(17:0_22:6)+HCOO	864.576011	C48 H83 O10 N1 P1	7.469
DG(16:0_22:6)+NH4	658.541	C41 H72 O5 N1	8.193	PC(17:0_22:6)+HCOO	864.576011	C48 H83 O10 N1 P1	7.581
DG(16:0_22:6)+NH4	658.541	C41 H72 O5 N1	8.356	PC(20:5_20:5)+HCOO	870.529061	C49 H77 O10 N1 P1	5.872
DG(18:0_22:6)+NH4	686.572	C43 H76 O5 N1	8.849	PC(18:0_22:6)+HCOO	878.591661	C49 H85 O10 N1 P1	7.912
DG(18:3_22:6)+NH4	680.525	C43 H70 O5 N1	7.5695	PC(18:0p_22:6)+HCOO	862.596746	C49 H85 O9 N1 P1	8.206
DG(20:4_22:6)+NH4	706.541	C45 H72 O5 N1	7.844	PC(18:1_22:6)+HCOO	876.576011	C49 H83 O10 N1 P1	7.328
DG(20:1_22:6)+NH4	712.587	C45 H78 O5 N1	8.8625	PC(20:5_20:4)+HCOO	872.544711	C49 H79 O10 N1 P1	6.339
DG(22:5_22:6)+NH4	732.556	C47 H74 O5 N1	7.651	PC(19:0_22:6)+HCOO	892.607311	C50 H87 O10 N1 P1	8.157
DG(14:0_20:5)+H	587.467	C37 H63 O5	7.223	PC(20:4_22:6)+HCOO	898.560361	C51 H81 O10 N1 P1	6.635
DG(20:5_18:2)+H	639.498	C41 H67 O5	7.752	PC(20:5_22:6)+HCOO	896.544711	C51 H79 O10 N1 P1	6.140
DG(18:3_20:5)+H	637.483	C41 H65 O5	7.2325	PC(20:0_22:6)+HCOO	906.622961	C51 H89 O10 N1 P1	8.541

DG(14:0_20:5)+NH4	604.494	C37 H66 O5 N1	7.867	PC(20:1_22:6)+HCOO	904.607311	C51 H87 O10 N1 P1	7.925
DG(16:0_20:5)+NH4	632.525	C39 H70 O5 N1	8.135	PC(20:2_22:6)+HCOO	902.591661	C51 H85 O10 N1 P1	7.363
DG(20:5_18:2)+NH4	656.525	C41 H70 O5 N1	7.7615	PC(22:5_22:6)+HCOO	924.576011	C53 H83 O10 N1 P1	6.702
DG(18:3_20:5)+NH4	654.509	C41 H68 O5 N1	7.2985	PC(22:6_22:6)+HCOO	922.560361	C53 H81 O10 N1 P1	6.446
DG(20:5_22:6)+H	687.498	C45 H67 O5	7.321	PE(22:6_22:6)-H	834.507931	C49 H73 O8 N1 P1	5.134
DG(20:5_22:6)+NH4	704.525	C45 H70 O5 N1	7.535	PE(22:6_22:6)-H	834.507931	C49 H73 O8 N1 P1	6.505
TG(18:0_22:6_22:6)+NH4	996.801	C65 H106 O6 N1	10.7595	PE(15:0_22:6)-H	748.492281	C42 H71 O8 N1 P1	6.944
TG(18:1_22:6_22:6)+NH4	994.786	C65 H104 O6 N1	10.5315	PE(16:0_22:6)-H	762.507931	C43 H73 O8 N1 P1	5.788
TG(18:2_22:6_22:6)+NH4	992.770	C65 H102 O6 N1	10.4185	PE(16:0_22:6)-H	762.507931	C43 H73 O8 N1 P1	7.029
TG(18:3_22:6_22:6)+NH4	990.755	C65 H100 O6 N1	10.211	PE(16:0e_22:6)-H	748.528666	C43 H75 O7 N1 P1	7.786
TG(20:1_22:6_22:6)+NH4	1022.817	C67 H108 O6 N1	10.875	PE(16:0p_22:6)-H	746.513016	C43 H73 O7 N1 P1	7.578
TG(20:4_22:6_22:6)+NH4	1016.770	C67 H102 O6 N1	10.334	PE(16:1_22:6)-H	760.492281	C43 H71 O8 N1 P1	6.689
TG(22:5_22:6_22:6)+NH4	1042.786	C69 H104 O6 N1	10.329	PE(38:7p)-H	744.497366	C43 H71 O7 N1 P1	7.127
TG(12:0_20:5_20:5)+NH4	860.676	C55 H90 O6 N1	9.7445	PE(17:0_22:6)-H	776.523581	C44 H75 O8 N1 P1	7.581
TG(14:0_20:5_20:5)+NH4	888.708	C57 H94 O6 N1	10.1405	PE(18:0_22:6)-H	790.539231	C45 H77 O8 N1 P1	7.868
TG(15:0_20:5_20:5)+NH4	902.723	C58 H96 O6 N1	10.258	PE(18:0p_22:6)-H	774.544316	C45 H77 O7 N1 P1	8.147
TG(20:5_18:2_20:5)+NH4	940.739	C61 H98 O6 N1	10.217	PE(18:1_22:6)-H	788.523581	C45 H75 O8 N1 P1	7.328
TG(18:3_20:5_20:5)+NH4	938.723	C61 H96 O6 N1	10.037	PE(18:1p_22:6)-H	772.528666	C45 H75 O7 N1 P1	7.627
TG(20:5_20:5_22:6)+NH4	988.739	C65 H98 O6 N1	9.9805	PE(18:2_22:6)-H	786.507931	C45 H73 O8 N1 P1	6.834
TG(22:6_22:6_22:6)+NH4	1040.770	C69 H102 O6 N1	10.205	PE(18:2p_22:6)-H	770.513016	C45 H73 O7 N1 P1	7.152
TG(20:5_20:5_20:5)+NH4	962.723	C63 H96 O6 N1	9.8645	PE(20:4_22:6)-H	810.507931	C47 H73 O8 N1 P1	6.690
TG(18:1_18:2_22:6)+H	929.759	C61 H101 O6	9.4965	PE(20:0p_22:6)-H	802.575616	C47 H81 O7 N1 P1	8.641
TG(4:0_16:0_22:6)+NH4	728.582	C45 H78 O6 N1	9.132	PE(20:2_22:6)-H	814.539231	C47 H77 O8 N1 P1	7.248
TG(6:0_16:0_22:6)+NH4	756.614	C47 H82 O6 N1	9.517	PE(20:2_22:6)-H	814.539231	C47 H77 O8 N1 P1	7.433
TG(16:0_8:0_22:6)+NH4	784.645	C49 H86 O6 N1	9.87	PE(22:6_21:1)-H	830.570531	C48 H81 O8 N1 P1	7.415
TG(14:0_14:0_22:6)+NH4	840.708	C53 H94 O6 N1	10.4055	PE(22:5_22:6)-H	836.523581	C49 H75 O8 N1 P1	6.742
TG(16:0_16:0_22:6)+NH4	896.770	C57 H102 O6 N1	10.822	PE(16:0_20:5)-H	736.492281	C41 H71 O8 N1 P1	7.012
TG(16:0_16:0_22:6)+NH4	896.770	C57 H102 O6 N1	10.9615	PE(16:0p_20:5)-H	720.497366	C41 H71 O7 N1 P1	7.332
TG(16:0_17:1_22:6)+NH4	908.770	C58 H102 O6 N1	10.877	PE(20:5_20:4)-H	784.492281	C45 H71 O8 N1 P1	6.404

TG(15:0_18:2_22:6)+NH4	906.755	C58 H100 O6 N1	10.6985	PE(20:5_22:6)-H	808.492281	C47 H71 O8 N1 P1	6.208
TG(16:0_18:1_22:6)+NH4	922.786	C59 H104 O6 N1	10.9575	PG(22:6_22:6)-H	865.502512	C50 H74 O10 N0 P1	5.220
TG(18:2_17:2_22:6)+NH4	930.755	C60 H100 O6 N1	10.548	PG(18:1_22:6)-H	819.518162	C46 H76 O10 N0 P1	6.029
TG(17:0_18:1_22:6)+NH4	936.801	C60 H106 O6 N1	11.1435	PG(18:2_22:6)-H	817.502512	C46 H74 O10 N0 P1	5.490
TG(18:2_18:2_22:6)+NH4	944.770	C61 H102 O6 N1	10.6325	PG(18:2_22:6)-H	817.502512	C46 H74 O10 N0 P1	5.897
TG(18:3_18:2_22:6)+NH4	942.755	C61 H100 O6 N1	10.4375	PG(20:4_22:6)-H	841.502512	C48 H74 O10 N0 P1	5.386
TG(18:0_18:0_22:6)+NH4	952.833	C61 H110 O6 N1	11.3825	PG(22:4_22:6)-H	869.533812	C50 H78 O10 N0 P1	5.924
TG(18:0_18:1_22:6)+NH4	950.817	C61 H108 O6 N1	11.1635	PG(22:5_22:6)-H	867.518162	C50 H76 O10 N0 P1	5.463
TG(18:1_18:1_22:6)+NH4	948.801	C61 H106 O6 N1	10.956	PG(20:5_22:6)-H	839.486862	C48 H72 O10 N0 P1	4.879
TG(18:0_20:4_22:6)+NH4	972.801	C63 H106 O6 N1	10.8835	PI(16:0_22:6)-H	881.518557	C47 H78 O13 N0 P1	6.127
TG(18:2_20:4_22:6)+NH4	968.770	C63 H102 O6 N1	10.545	PI(17:0_22:6)-H	895.534207	C48 H80 O13 N0 P1	6.473
TG(20:1_18:1_22:6)+NH4	976.833	C63 H110 O6 N1	11.332	PI(18:0_22:6)-H	909.549857	C49 H82 O13 N0 P1	6.795
TG(18:2_21:5_22:6)+NH4	980.770	C64 H102 O6 N1	10.4125	PI(18:1_22:6)-H	907.534207	C49 H80 O13 N0 P1	6.214
TG(16:0_22:6_23:1)+NH4	992.864	C64 H114 O6 N1	11.6065	PI(16:0_20:5)-H	855.502907	C45 H76 O13 N0 P1	5.827
TG(18:1_22:4_22:6)+NH4	998.817	C65 H108 O6 N1	10.948	PI(18:0_20:5)-H	883.534207	C47 H80 O13 N0 P1	6.794
TG(16:0_22:6_24:1)+NH4	1006.880	C65 H116 O6 N1	11.7585	PS(22:6_22:6)-H	878.497761	C50 H73 O10 N1 P1	6.693
TG(18:1_22:1_22:6)+NH4	1004.864	C65 H114 O6 N1	11.5525	PS(16:0_22:6)-H	806.497761	C44 H73 O10 N1 P1	6.435
TG(22:1_18:2_22:6)+NH4	1002.848	C65 H112 O6 N1	11.3615	PS(16:0_22:6)-H	806.497761	C44 H73 O10 N1 P1	7.076
TG(22:5_20:4_22:6)+NH4	1018.786	C67 H104 O6 N1	10.4495	PS(18:0_22:6)-H	834.529061	C46 H77 O10 N1 P1	7.087
TG(18:0_22:6_24:1)+NH4	1034.911	C67 H120 O6 N1	11.955	PS(18:0_22:6)-H	834.529061	C46 H77 O10 N1 P1	8.004
TG(18:1_22:6_24:1)+NH4	1032.895	C67 H118 O6 N1	11.7725	PS(18:1_22:6)-H	832.513411	C46 H75 O10 N1 P1	7.495
TG(15:0_14:0_20:5)+NH4	828.708	C52 H94 O6 N1	10.492	PS(20:4_22:6)-H	854.497761	C48 H73 O10 N1 P1	6.835
