# Supplementary material for: Endurance Training Counteracts the High-Fat Diet-Induced Profiling Changes of ω-3 Polyunsaturated Fatty Acids in Skeletal Muscle of Middle-Aged Rats
Source: Front Physiol. 2019 Jul 30;10:971. doi: 10.3389/fphys.2019.00971 (PMC6683664; doi:10.3389/fphys.2019.00971)
Supplement: Supplementary file 2 [file Table_2.pdf]

Table 2: VIP values of PLS-DA, fold-change and p values of *t*-test: values of VIP > 1.0, $|Log_2(FC)| > 1.5$ and $p < 0.05$ are highlighted.

	ALL	C vs EC			C vs H			EC vs EH			H vs EH		
	VIP	VIP	$LOG_2(FC)$	P VALUE	VIP	$LOG_2(FC)$	P VALUE	VIP	LOG2(FC)	P VALUE	VIP	$LOG_2(FC)$	P VALUE
CL 18:2_18:2_18:2_22:6	0.338	1.06	-0.549	0.000	0.442	-0.392	0.000	0.511	0.193	0.002	0.055	0.036	0.672
CL 20:5_16:0_16:0_18:2	0.201	0.608	-0.049	0.000	0.262	-0.130	0.005	0.734	0.413	0.000	0.492	0.494	0.000
CL 20:5_16:0_18:1_16:0	0.297	0.238	0.366	0.004	0.067	0.371	0.160	0.455	0.101	0.000	0.136	0.095	0.029
CL 20:5_16:1_18:2_16:1	0.34	0.796	-0.234	0.000	0.422	-0.359	0.000	0.327	-0.055	0.025	0.104	0.070	0.179
CL 20:5_16:1_18:2_18:2	0.447	0.919	-0.387	0.000	0.642	-0.695	0.000	0.386	0.025	0.014	0.336	0.332	0.009
CL 20:5_18:1_16:1_18:2	1.458	1.051	-0.534	0.000	1.077	-1.343	0.000	0.459	-0.943	0.001	0.074	-0.134	0.234
CL 20:5_20:4_18:2_18:2	0.392	0.048	0.585	0.558	0.263	-0.121	0.000	0.288	-0.747	0.008	0.011	-0.041	0.882
DG 14:0_20:5	2.493	0.333	0.245	0.010	0.188	0.534	0.043	2.898	2.858	0.000	2.368	2.569	0.000
DG 14:0_22:6	0.393	1.626	-1.156	0.000	1.27	-1.600	0.000	1.182	0.908	0.000	1.282	1.352	0.000
DG 16:0_20:5	1.152	0.486	0.088	0.000	0.009	0.289	0.880	0.964	-1.498	0.000	1.479	-1.700	0.000
DG 16:0_22:6	0.327	0.916	-0.356	0.000	0.362	-0.252	0.002	0.394	-0.002	0.008	0.028	-0.106	0.809
DG 18:0_22:6	0.806	1.323	-0.802	0.000	0.569	-0.543	0.003	0.219	-0.219	0.210	0.335	-0.478	0.073
DG 20:1_22:6	1.276	0.556	0.036	0.000	0.349	-0.241	0.000	1.93	1.745	0.000	1.879	2.022	0.000
DG 20:4_22:6	0.976	0.286	0.931	0.009	0.767	-0.885	0.000	0.932	-1.467	0.000	0.363	0.348	0.000
DG 20:5_18:2	1.945	0.821	1.536	0.000	0.797	-0.895	0.000	2.342	-3.037	0.000	0.499	-0.606	0.003
DG 22:5_22:6	0.449	0.619	-0.051	0.000	0.079	0.162	0.227	0.059	-0.483	0.475	0.571	-0.697	0.000
DG 22:6_22:6	0.169	0.517	1.197	0.000	0.494	1.002	0.000	0.367	-0.826	0.000	0.519	-0.631	0.000
FA 20:5	0.548	0.504	0.067	0.000	0.483	-0.447	0.000	0.067	-0.491	0.097	0.075	0.023	0.260
FA 22:6	0.373	0.912	-0.376	0.000	0.545	-0.543	0.000	0.421	0.070	0.003	0.249	0.237	0.028
LPC 20:5	0.102	0.293	0.950	0.004	0.075	0.154	0.172	0.272	-0.711	0.009	0.116	0.085	0.155
LPC 22:6	0.014	0.041	0.666	0.640	0.046	0.198	0.569	0.009	-0.401	0.855	0.111	0.066	0.182
LPE 20:5	1.299	0.864	-0.314	0.000	1.137	-1.410	0.000	0.378	-0.856	0.001	0.275	0.240	0.003
LPE 22:6	0.065	1.035	-0.500	0.000	0.349	-0.245	0.000	0.9	0.589	0.000	0.352	0.334	0.000

LPI 22:6	0.515	0.758	-0.221	0.000	0.031	0.303	0.637	1.052	0.772	0.000	0.274	0.248	0.000
LPS 22:6	0.507	1.023	-0.499	0.000	1.212	-1.526	0.000	0.637	0.304	0.000	1.25	1.331	0.000
MG 22:6	0.553	0.468	0.106	0.000	0.091	0.127	0.054	0.959	0.670	0.000	0.632	0.649	0.000
PA 24:2_22:6	0.721	1.47	-0.955	0.000	0.21	0.583	0.000	1.667	1.429	0.000	0.05	-0.109	0.376
PAF 20:5	0.562	0.43	0.156	0.000	0.492	-0.456	0.000	0.126	-0.551	0.060	0.102	0.060	0.162
PC 14:0_20:5	0.592	0.644	-0.082	0.000	0.452	-0.388	0.000	0.038	-0.458	0.459	0.076	-0.152	0.345
PC 14:0_22:6	0.307	0.105	0.511	0.349	0.246	-0.102	0.003	0.164	-0.581	0.227	0.057	0.033	0.625
PC 14:0e_22:6	0.09	0.092	0.733	0.340	0.196	-0.017	0.016	0.073	-0.502	0.361	0.28	0.248	0.004
PC 15:0_22:6	0.057	0.184	0.427	0.136	0.195	-0.015	0.051	0.142	-0.264	0.131	0.223	0.178	0.040
PC 16:0_22:6	0.649	1.299	-0.812	0.000	0.835	-0.923	0.000	0.495	0.159	0.002	0.326	0.270	0.117
PC 16:0e_22:6	0.506	0.673	-0.109	0.000	0.331	-0.209	0.000	0.024	-0.392	0.764	0.204	-0.292	0.043
PC 16:0p_22:6	0.443	0.025	0.654	0.737	0.097	0.125	0.093	0.465	-0.942	0.000	0.319	-0.413	0.000
PC 16:1_22:6	0.282	0.064	0.694	0.331	0.429	0.902	0.000	0.082	-0.310	0.258	0.426	-0.517	0.000
PC 17:0_22:6	0.298	0.565	0.007	0.000	0.222	-0.039	0.011	0.136	-0.258	0.084	0.128	-0.213	0.250
PC 18:0_22:6	0.116	0.647	-0.093	0.000	0.314	-0.198	0.000	0.431	0.087	0.001	0.215	0.192	0.079
PC 18:0p_22:6	0.315	0.407	0.186	0.002	0.293	-0.172	0.000	0.045	-0.365	0.640	0.033	-0.007	0.574
PC 18:1_22:6	0.056	0.832	-0.303	0.000	0.01	0.257	0.918	0.601	0.255	0.000	0.231	-0.306	0.005
PC 19:0_22:6	0.929	0.812	-0.245	0.000	0.185	-0.008	0.002	0.419	-0.916	0.003	0.989	-1.153	0.000
PC 20:0_22:6	0.059	0.565	0.005	0.000	0.148	0.053	0.004	0.499	0.155	0.000	0.144	0.107	0.111
PC 20:1_22:6	0.504	0.034	0.666	0.540	0.485	0.991	0.000	0.324	-0.043	0.001	0.284	-0.368	0.003
PC 20:2_22:6	0.292	0.529	0.026	0.003	0.443	-0.412	0.001	0.215	-0.178	0.010	0.285	0.260	0.000
PC 20:4_22:6	0.007	0.409	1.080	0.000	0.055	0.192	0.351	0.25	-0.699	0.001	0.227	0.188	0.005
PC 20:5_20:4	0.617	0.508	0.083	0.000	0.676	-0.725	0.000	0.056	-0.473	0.654	0.34	0.335	0.008
PC 20:5_20:5	0.278	0.308	0.288	0.012	0.315	-0.194	0.003	0.031	-0.390	0.679	0.147	0.092	0.108
PC 20:5_22:6	2.993	3.023	3.954	0.000	2.451	3.892	0.000	0.27	-0.107	0.000	0.008	-0.045	0.678
PC22:5_22:6	0.358	0.917	-0.385	0.000	0.714	-0.788	0.000	0.514	0.175	0.000	0.565	0.578	0.000
PC 22:6_22:6	0.35	1.021	-0.486	0.000	0.402	-0.324	0.000	0.454	0.082	0.001	0.018	-0.080	0.823

PE 15:0_22:6	0.319	0.205	0.410	0.054	0.198	0.564	0.001	0.401	0.030	0.001	0.061	-0.124	0.293
PE 16:0_20:5	0.448	0.524	0.053	0.000	0.728	-0.811	0.000	0.164	-0.232	0.089	0.62	0.631	0.000
PE 16:0_22:6	2.68	0.29	0.946	0.003	1.723	-2.294	0.000	2.405	-3.126	0.000	0.147	0.114	0.115
PE 16:0e_22:6	0.144	0.924	-0.387	0.000	0.089	0.138	0.195	0.801	0.482	0.000	0.015	-0.043	0.861
PE 16:0p_20:5	0.368	0.571	-0.006	0.000	0.114	0.107	0.087	0.014	-0.359	0.915	0.403	-0.472	0.020
PE 16:0p_22:6	0.544	0.58	0.000	0.000	0.344	-0.234	0.000	0.074	-0.503	0.543	0.192	-0.269	0.107
PE 16:1_22:6	0.002	0.545	0.034	0.000	0.073	0.173	0.202	0.386	0.015	0.000	0.054	-0.123	0.458
PE 16:1p_22:6	0.138	0.313	0.283	0.000	0.075	0.162	0.158	0.39	0.035	0.000	0.187	0.156	0.062
PE 17:0_22:6	0.294	1.193	-0.660	0.000	0.381	-0.284	0.005	1.272	0.978	0.000	0.617	0.602	0.001
PE18:0_22:6	1.103	3.621	4.625	0.000	1.285	2.171	0.000	1.704	-2.337	0.000	0.154	0.117	0.000
PE 18:0p_22:6	0.489	0.45	0.130	0.000	0.148	0.497	0.018	0.771	0.464	0.000	0.139	0.097	0.175
PE 18:1_22:6	0.504	2.313	3.187	0.000	3.071	4.830	0.000	2.297	-3.009	0.000	4.143	-4.652	0.000
PE 18:1p_22:6	0.128	0.612	-0.051	0.000	0.353	-0.259	0.000	0.413	0.051	0.000	0.282	0.260	0.000
PE 18:2_22:6	1.149	1.334	2.096	0.000	0.119	0.091	0.096	0.455	0.099	0.000	1.952	2.104	0.000
PE 18:2p_22:6	0.28	0.202	0.405	0.106	0.199	0.003	0.104	0.091	-0.514	0.446	0.029	-0.112	0.853
PE 20:0p_22:6	2.145	1.511	-1.040	0.000	2.791	-3.837	0.000	0.168	-0.603	0.149	2.058	2.194	0.000
PE 20:2_22:6	0.58	0.99	-0.474	0.000	0.531	-0.495	0.000	0.236	-0.137	0.039	0.036	-0.116	0.823
PE 20:4_22:6	0.236	0.701	-0.151	0.000	0.294	-0.161	0.000	0.325	-0.036	0.001	0.025	-0.026	0.794
PE 20:5_20:4	0.08	0.67	-0.115	0.000	0.406	-0.330	0.000	0.526	0.188	0.000	0.412	0.403	0.000
PE 20:5_22:6	0.337	0.92	-0.388	0.000	0.511	-0.479	0.000	0.451	0.106	0.000	0.226	0.196	0.047
PE 22:5_22:6	0.46	0.212	0.395	0.044	0.671	-0.700	0.000	0.078	-0.502	0.401	0.608	0.593	0.001
PE 22:6_21:1	1.485	0.648	1.330	0.000	1.19	-1.427	0.000	1.548	-2.161	0.000	0.664	0.596	0.010
PE 22:6_22:6	0.884	1.197	-0.687	0.000	0.8	-0.922	0.000	0.152	-0.253	0.155	0.033	-0.018	0.592
PG 18:1_22:6	0.187	0.243	0.351	0.031	0.114	0.428	0.138	0.316	-0.049	0.008	0.072	-0.127	0.467
PG 18:2_22:6	0.098	0.029	0.586	0.698	0.274	0.666	0.000	0.008	-0.392	0.915	0.388	-0.473	0.000
PG 20:4_22:6	0.144	0.531	0.020	0.001	0.813	1.457	0.000	0.169	-0.582	0.150	1.789	-2.019	0.000
PG 20:5_22:6	0.116	0.024	0.636	0.855	0.139	0.457	0.187	0.052	-0.357	0.464	0.109	-0.178	0.203

PG 22:4_22:6	1.039	0.071	0.536	0.582	0.726	-0.787	0.000	0.787	-1.296	0.000	0.1	0.026	0.569
PG 22:5_22:6	0.181	0.349	0.227	0.013	0.142	0.459	0.121	0.366	0.001	0.001	0.164	-0.232	0.054
PI 16:0_20:5	0.576	0.505	0.063	0.000	1.519	-1.996	0.000	0.357	-0.004	0.001	1.893	2.055	0.000
PI 16:0_22:6	0.798	0.374	0.218	0.000	0.333	0.768	0.000	0.983	0.694	0.000	0.182	0.144	0.008
PI 17:0_22:6	0.035	0.43	0.165	0.001	0.318	-0.198	0.000	0.459	0.107	0.003	0.465	0.470	0.001
PI 18:0_20:5	0.292	0.614	-0.037	0.000	0.007	0.290	0.889	0.722	0.392	0.000	0.114	0.065	0.183
PI 18:0_22:6	0.256	3.149	-2.819	0.000	0.079	0.152	0.001	1.807	1.604	0.000	1.194	-1.367	0.000
PI 18:1_22:6	0.068	0.706	-0.149	0.000	0.262	-0.113	0.000	0.651	0.322	0.000	0.31	0.286	0.001
PS 16:0_22:6	0.866	0.79	-0.247	0.000	0.358	0.789	0.000	1.319	1.070	0.000	0.077	0.035	0.137
PS 18:0_226	0.459	0.668	-0.105	0.000	0.653	-0.706	0.000	0.214	-0.180	0.019	0.425	0.421	0.000
PS 20:4_22:6	0.007	0.321	0.273	0.001	0.168	0.016	0.001	0.291	-0.071	0.010	0.201	0.186	0.032
PS 22:6_22:6	0.093	0.415	0.179	0.001	0.066	0.182	0.326	0.403	0.032	0.001	0.085	0.028	0.338
TG 14:0_20:5_20:5	1.639	0.927	-0.402	0.000	0.777	-0.899	0.000	0.871	-1.015	0.100	0.771	-0.518	0.143
TG 15:0_18:2_22:6	0.891	0.099	0.517	0.041	0.778	-0.888	0.000	0.582	-1.057	0.000	0.35	0.348	0.001
TG 15:0_20:5_20:5	2	0.385	1.036	0.005	3.756	-3.126	0.008	0.822	-1.337	0.000	4.574	2.825	0.014
TG 15:0_20:5_22:6	1.282	0.77	-0.215	0.000	2.403	-2.985	0.000	0.134	-0.271	0.159	2.585	2.499	0.000
TG 16:0_14:0_20:5	2.498	6.084	-5.777	0.000	0.724	1.347	0.000	6.422	6.518	0.000	0.496	-0.606	0.000
TG 16:0_16:0_22:6	0.846	0.187	0.826	0.024	0.653	-0.698	0.000	0.774	-1.266	0.000	0.272	0.259	0.038
TG 16:0_18:1_22:6	0.628	0.506	0.066	0.000	0.641	-0.676	0.000	0.085	-0.488	0.387	0.267	0.255	0.038
TG 16:0_22:6_24:1	0.595	0.094	0.510	0.340	1.118	-1.363	0.000	0.108	-0.516	0.278	1.293	1.357	0.000
TG 16:0_8:0_22:6	0.635	0.711	-0.136	0.001	1.156	-1.450	0.000	0.266	-0.146	0.054	1.11	1.168	0.000
TG 16:2_14:0_20:5	0.624	0.445	0.133	0.002	0.821	1.475	0.000	0.76	-1.176	0.007	2.317	-2.519	0.000
TG 17:0_18:1_22:6	0.005	0.253	0.352	0.000	0.371	-0.264	0.000	0.322	-0.044	0.001	0.573	0.572	0.000
TG 17:0_20:5_22:6	1.933	0.334	0.263	0.001	2.446	-3.099	0.000	0.853	-1.357	0.000	2.073	2.005	0.001
TG 18:0_18:1_22:6	0.579	0.482	0.077	0.002	0.523	-0.494	0.002	0.098	-0.520	0.278	0.119	0.051	0.430
TG 18:0_20:4_22:6	0.895	0.61	-0.054	0.000	1.187	-1.483	0.000	0.074	-0.494	0.407	0.905	0.936	0.000
TG 18:0_22:6_22:6	1.321	0.298	0.946	0.004	1.626	-1.987	0.000	0.946	-1.481	0.000	1.51	1.452	0.000

TG 18:0_22:6_24:1	0.929	0.788	-0.233	0.000	1.337	-1.657	0.000	0.07	-0.349	0.470	1.076	1.075	0.000
TG 18:1_18:1_22:6	0.881	0.315	0.283	0.000	1.037	-1.256	0.000	0.316	-0.740	0.024	0.752	0.799	0.000
TG 18:1_22:4_22:6	1.164	0.477	0.099	0.003	1.814	-1.980	0.000	0.184	-0.622	0.104	1.765	1.456	0.003
TG 18:1_22:6_22:6	1.18	0.342	0.250	0.001	1.919	-2.521	0.000	0.244	-0.683	0.011	1.979	2.089	0.000
TG 18:2_17:2_22:6	1.812	0.372	0.227	0.002	1.483	-1.751	0.000	1.116	-1.655	0.000	0.477	0.322	0.149
TG 18:2_18:2_22:6	1.258	0.138	0.481	0.079	1.3	-1.647	0.000	0.735	-1.237	0.000	0.855	0.891	0.000
TG 18:2_20:4_22:6	1.728	0.22	0.370	0.033	1.561	-2.038	0.000	1.088	-1.621	0.000	0.762	0.787	0.000
TG 18:2_22:6_22:6	1.625	0.133	0.753	0.206	1.939	-2.601	0.000	1.038	-1.572	0.000	1.67	1.782	0.000
TG 18:3_18:2_20:5	0.649	0.111	0.498	0.352	0.641	-0.695	0.000	0.366	-0.813	0.009	0.374	0.380	0.004
TG 18:3_18:2_22:6	2.237	0.302	0.304	0.016	1.449	-1.885	0.000	1.647	-2.140	0.000	0.038	0.050	0.893
TG 18:3_20:5_20:5	1.476	0.242	0.374	0.025	0.561	-0.558	0.000	1.232	-1.731	0.000	0.745	-0.799	0.003
TG 18:3_20:5_22:6	1.957	0.47	1.134	0.001	0.233	0.608	0.010	1.761	1.575	0.000	1.949	2.102	0.000
TG 18:4_14:0_20:5	0.96	0.952	-0.429	0.000	1.021	-1.230	0.000	0.004	-0.410	0.969	0.422	0.391	0.018
TG 18:4_18:2_20:5	0.983	0.142	0.462	0.156	0.412	-0.343	0.001	0.816	-1.328	0.000	0.422	-0.522	0.007
TG 20:5_14:1_18:2	0.797	0.659	-0.101	0.000	0.8	-0.933	0.000	0.103	-0.501	0.545	0.309	0.331	0.059
TG 20:5_17:1_22:6	2.337	0.542	0.003	0.002	2.75	-3.806	0.000	1.032	-1.573	0.000	2.091	2.237	0.000
TG 20:5_18:2_18:2	1.295	0.271	0.335	0.001	1.051	-1.291	0.000	0.798	-1.312	0.000	0.323	0.313	0.000
TG 20:5_18:2_20:5	1.468	0.723	-0.141	0.000	1.101	-1.364	0.000	0.672	-1.125	0.009	0.069	0.098	0.718
TG 20:5_18:2_22:6	1.762	0.119	0.496	0.086	1.96	-2.629	0.000	1.017	-1.539	0.000	1.47	1.586	0.000
TG 4:0_16:0_22:6	0.977	0.417	1.079	0.000	0.441	-0.382	0.000	1.162	-1.714	0.000	0.177	-0.252	0.133
TG 6:0_16:0_22:6	1.279	0.225	0.870	0.003	0.878	-1.037	0.000	1.181	-1.694	0.000	0.198	0.213	0.240
