# Supplementary material for: Endurance Training Counteracts the High-Fat Diet-Induced Profiling Changes of ω-3 Polyunsaturated Fatty Acids in Skeletal Muscle of Middle-Aged Rats
Source: Front Physiol. 2019 Jul 30;10:971. doi: 10.3389/fphys.2019.00971 (PMC6683664; doi:10.3389/fphys.2019.00971)

Figure 1: Heatmap of top 25% lipid species in the skeletal muscle of the rats: C, chow diet; E, Endurance training with chow diet; H, high-fat diet; HE, endurance training with high-fat diet

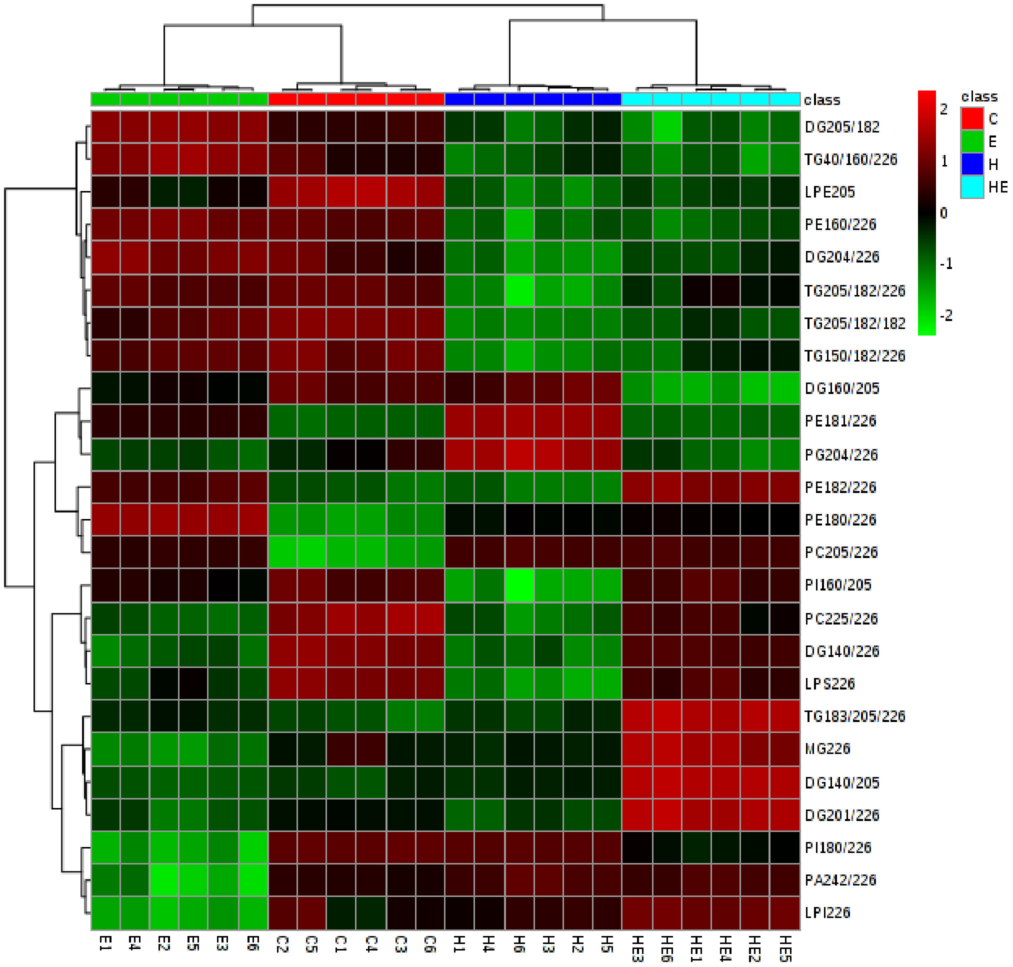

Supplement: Supplementary file 3 [file Image_1.pdf]
